# Supplementary material for: Modeling glioblastoma heterogeneity as a dynamic network of cell states
Source: Mol Syst Biol. 2021 Sep 16;17(9):e10105. doi: 10.15252/msb.202010105 (PMC8444284; doi:10.15252/msb.202010105)
Supplement: Supplementary file 5 — Source Data for Figure 3 [file MSB-17-e10105-s001.zip › Figure3A_sourcedata/GSEA_3065/nosigdb_state6.GseaPreranked.1622718443993/LARSSON_STATE_5.html]

Details for gene set LARSSON\_STATE\_5[GSEA]

|  || Dataset | state6 |
| Phenotype | NoPhenotypeAvailable |
| Upregulated in class | na\_neg |
| GeneSet | LARSSON\_STATE\_5 |
| Enrichment Score (ES) | -0.56440693 |
| Normalized Enrichment Score (NES) | -2.2362978 |
| Nominal p-value | 0.0 |
| FDR q-value | 0.0 |
| FWER p-Value | 0.0 |
Table: GSEA Results Summary

  

Fig 1: Enrichment plot: LARSSON\_STATE\_5      
 Profile of the Running ES Score & Positions of GeneSet Members on the Rank Ordered List

  

| PROBE | GENE SYMBOL | GENE\_TITLE | RANK IN GENE LIST | RANK METRIC SCORE | RUNNING ES | CORE ENRICHMENT || 1 | HMOX1 |  |  | 0 | 0.545 | 0.0190 | No |
| 2 | CDKN1A |  |  | 1 | 0.477 | 0.0356 | No |
| 3 | GADD45A |  |  | 2 | 0.419 | 0.0503 | No |
| 4 | SOX4 |  |  | 3 | 0.375 | 0.0634 | No |
| 5 | BTG1 |  |  | 4 | 0.348 | 0.0755 | No |
| 6 | TMSB10 |  |  | 5 | 0.330 | 0.0870 | No |
| 7 | RPLP1 |  |  | 6 | 0.329 | 0.0985 | No |
| 8 | PCBP4 |  |  | 7 | 0.329 | 0.1099 | No |
| 9 | ENHO |  |  | 8 | 0.328 | 0.1214 | No |
| 10 | EEF1A1 |  |  | 9 | 0.325 | 0.1327 | No |
| 11 | RPLP0 |  |  | 10 | 0.312 | 0.1436 | No |
| 12 | RPS27 |  |  | 11 | 0.307 | 0.1543 | No |
| 13 | RPL11 |  |  | 12 | 0.302 | 0.1648 | No |
| 14 | C9orf16 |  |  | 13 | 0.300 | 0.1753 | No |
| 15 | RPS19 |  |  | 14 | 0.295 | 0.1856 | No |
| 16 | PABPC1 |  |  | 15 | 0.292 | 0.1958 | No |
| 17 | RPL37A |  |  | 16 | 0.290 | 0.2059 | No |
| 18 | TSHZ1 |  |  | 17 | 0.289 | 0.2159 | No |
| 19 | CNP |  |  | 18 | 0.288 | 0.2260 | No |
| 20 | RPL37 |  |  | 19 | 0.287 | 0.2360 | No |
| 21 | EEF2 |  |  | 20 | 0.286 | 0.2460 | No |
| 22 | RPL13 |  |  | 21 | 0.286 | 0.2560 | No |
| 23 | RPL3 |  |  | 22 | 0.285 | 0.2659 | No |
| 24 | RAB31 |  |  | 23 | 0.284 | 0.2758 | No |
| 25 | RPS28 |  |  | 24 | 0.283 | 0.2857 | No |
| 26 | RPL22 |  |  | 26 | 0.274 | 0.2951 | No |
| 27 | PPP1R14B |  |  | 27 | 0.274 | 0.3047 | No |
| 28 | ZFAS1 |  |  | 28 | 0.273 | 0.3142 | No |
| 29 | PHLDA3 |  |  | 29 | 0.272 | 0.3237 | No |
| 30 | HOMER3 |  |  | 30 | 0.271 | 0.3332 | No |
| 31 | RPLP2 |  |  | 31 | 0.271 | 0.3426 | No |
| 32 | CCND2 |  |  | 32 | 0.269 | 0.3520 | No |
| 33 | RPL10 |  |  | 33 | 0.265 | 0.3612 | No |
| 34 | RPL30 |  |  | 34 | 0.260 | 0.3703 | No |
| 35 | SARS |  |  | 35 | 0.260 | 0.3794 | No |
| 36 | RPL18 |  |  | 36 | 0.256 | 0.3883 | No |
| 37 | NDUFC1 |  |  | 37 | 0.255 | 0.3972 | No |
| 38 | RPL6 |  |  | 38 | 0.254 | 0.4061 | No |
| 39 | RPL18A |  |  | 39 | 0.252 | 0.4149 | No |
| 40 | ZMAT3 |  |  | 40 | 0.252 | 0.4236 | No |
| 41 | RPL34 |  |  | 41 | 0.251 | 0.4324 | No |
| 42 | PDIA4 |  |  | 8712 | -0.251 | -0.5557 | Yes |
| 43 | SNTG1 |  |  | 8713 | -0.251 | -0.5469 | Yes |
| 44 | CLN5 |  |  | 8714 | -0.253 | -0.5381 | Yes |
| 45 | CTGF |  |  | 8715 | -0.254 | -0.5292 | Yes |
| 46 | RPN2 |  |  | 8716 | -0.256 | -0.5203 | Yes |
| 47 | CPD |  |  | 8717 | -0.256 | -0.5114 | Yes |
| 48 | B2M |  |  | 8718 | -0.258 | -0.5024 | Yes |
| 49 | IGFBP2 |  |  | 8719 | -0.260 | -0.4933 | Yes |
| 50 | HELLS |  |  | 8720 | -0.260 | -0.4843 | Yes |
| 51 | EPRS |  |  | 8721 | -0.260 | -0.4752 | Yes |
| 52 | PRH1 |  |  | 8722 | -0.260 | -0.4661 | Yes |
| 53 | TMEM59 |  |  | 8723 | -0.261 | -0.4570 | Yes |
| 54 | SYPL1 |  |  | 8724 | -0.261 | -0.4479 | Yes |
| 55 | NQO1 |  |  | 8725 | -0.262 | -0.4387 | Yes |
| 56 | PTPRG |  |  | 8726 | -0.263 | -0.4295 | Yes |
| 57 | MCAM |  |  | 8727 | -0.266 | -0.4203 | Yes |
| 58 | CD46 |  |  | 8728 | -0.267 | -0.4110 | Yes |
| 59 | TEX264 |  |  | 8729 | -0.267 | -0.4017 | Yes |
| 60 | LAPTM4A |  |  | 8730 | -0.267 | -0.3923 | Yes |
| 61 | FUS |  |  | 8731 | -0.268 | -0.3830 | Yes |
| 62 | DNM1 |  |  | 8732 | -0.268 | -0.3736 | Yes |
| 63 | FAF2 |  |  | 8733 | -0.269 | -0.3643 | Yes |
| 64 | CENPE |  |  | 8734 | -0.270 | -0.3548 | Yes |
| 65 | CANX |  |  | 8735 | -0.270 | -0.3454 | Yes |
| 66 | RPN1 |  |  | 8736 | -0.272 | -0.3359 | Yes |
| 67 | PNISR |  |  | 8737 | -0.273 | -0.3264 | Yes |
| 68 | TMX1 |  |  | 8738 | -0.275 | -0.3168 | Yes |
| 69 | CCK |  |  | 8739 | -0.275 | -0.3072 | Yes |
| 70 | GGA1 |  |  | 8740 | -0.278 | -0.2975 | Yes |
| 71 | ANXA2 |  |  | 8741 | -0.279 | -0.2878 | Yes |
| 72 | KCNQ1OT1 |  |  | 8742 | -0.281 | -0.2780 | Yes |
| 73 | GABBR2 |  |  | 8743 | -0.281 | -0.2682 | Yes |
| 74 | LGALS3BP |  |  | 8744 | -0.282 | -0.2583 | Yes |
| 75 | BCAP29 |  |  | 8745 | -0.284 | -0.2484 | Yes |
| 76 | SRPX |  |  | 8746 | -0.285 | -0.2385 | Yes |
| 77 | ADGRV1 |  |  | 8747 | -0.285 | -0.2286 | Yes |
| 78 | UBE2C |  |  | 8748 | -0.286 | -0.2186 | Yes |
| 79 | DKK1 |  |  | 8749 | -0.288 | -0.2086 | Yes |
| 80 | LENG8 |  |  | 8750 | -0.289 | -0.1985 | Yes |
| 81 | TIMP1 |  |  | 8751 | -0.290 | -0.1884 | Yes |
| 82 | TFPI2 |  |  | 8752 | -0.290 | -0.1783 | Yes |
| 83 | MDM4 |  |  | 8753 | -0.290 | -0.1682 | Yes |
| 84 | KDELR1 |  |  | 8754 | -0.292 | -0.1580 | Yes |
| 85 | APLP2 |  |  | 8755 | -0.292 | -0.1478 | Yes |
| 86 | ADAM9 |  |  | 8756 | -0.296 | -0.1375 | Yes |
| 87 | APP |  |  | 8758 | -0.300 | -0.1271 | Yes |
| 88 | HMGCR |  |  | 8759 | -0.301 | -0.1166 | Yes |
| 89 | VMP1 |  |  | 8760 | -0.302 | -0.1061 | Yes |
| 90 | DYNC1H1 |  |  | 8761 | -0.302 | -0.0956 | Yes |
| 91 | CD9 |  |  | 8762 | -0.303 | -0.0850 | Yes |
| 92 | SPCS2 |  |  | 8763 | -0.307 | -0.0743 | Yes |
| 93 | SMC4 |  |  | 8764 | -0.307 | -0.0636 | Yes |
| 94 | TMED9 |  |  | 8765 | -0.311 | -0.0528 | Yes |
| 95 | NKTR |  |  | 8766 | -0.315 | -0.0418 | Yes |
| 96 | SQLE |  |  | 8767 | -0.319 | -0.0306 | Yes |
| 97 | RRBP1 |  |  | 8768 | -0.320 | -0.0194 | Yes |
| 98 | TECR |  |  | 8769 | -0.321 | -0.0082 | Yes |
| 99 | CHST12 |  |  | 8770 | -0.322 | 0.0030 | Yes |
Table: GSEA details [plain text format]

  

Fig 2: LARSSON\_STATE\_5: Random ES distribution      
 Gene set null distribution of ES for **LARSSON\_STATE\_5**
